# From refugia to contact: Pine processionary moth hybrid zone in a complex biogeographic setting

**DOI:** 10.1002/ece3.6018

**Published:** 2020-01-28

**Authors:** Kahraman İpekdal, Christian Burban, Laure Sauné, Andrea Battisti, Carole Kerdelhué

**Affiliations:** ^1^ Faculty of Agriculture Ahi Evran University Kırşehir Turkey; ^2^ INRAE, BIOGECO (INRAE, Univ. Bordeaux) Cestas France; ^3^ INRAE, CBGP (INRAE, CIRAD, RD, Montpellier Supagro, Univ. Montpellier) Montpellier France; ^4^ DAFNAE‐Entomology University of Padua Legnaro Italy

**Keywords:** Aegean Sea, asymmetric introgression, natural hybridization, secondary contact, *Thaumetopoea pityocampa*, *Thaumetopoea wilkinsoni*

## Abstract

Contact zones occur at the crossroad between specific dispersal routes and are facilitated by biogeographic discontinuities. Here, we focused on two Lepidoptera sister species that come in contact near the Turkish Straits System (TSS). We aimed to infer their phylogeographic histories in the Eastern Mediterranean and finely analyze their co‐occurrence and hybridization patterns in this biogeographic context.

We used molecular mitochondrial and nuclear markers to study 224 individuals from 42 localities. We used discordances between markers and complementary assignment methods to identify and map hybrids and parental individuals.

We confirmed the parapatric distribution of *Thaumetopoea pityocampa* (Lepidoptera: Notodontidae) in the west and *Thaumetopoea wilkinsoni* in the east and identified a narrow contact zone. We identified several glacial refugia of *T. wilkinsoni* in southern Turkey with a strong east–west differentiation in this species. Unexpectedly, *T. pityocampa* crossed the TSS and occur in northern Aegean Turkey and some eastern Greek islands. We found robust evidence of introgression between the two species in a restricted zone in northwestern Turkey, but we did not identify any F_1_ individuals. The identified hybrid zone was mostly bimodal.

The distributions and genetic patterns of the studied species were strongly influenced both by the Quaternary climatic oscillations and the complex geological history of the Aegean region. *T. pityocampa* and *T. wilkinsoni* survived the last glacial maximum in disjoint refugia and met in western Turkey at the edge of the recolonization routes. Expanding population of *T. wilkinsoni* constrained *T. pityocampa* to the western Turkish shore. Additionally, we found evidence of recurrent introgression by *T. wilkinsoni* males in several *T. pityocampa* populations. Our results suggest that some prezygotic isolation mechanisms, such as differences in timing of the adult emergences, might be a driver of the isolation between the sister species.

## Graphical abstract

In this study, we show evidences of natural hybridization between two pine processionary moth species in Turkey by using mitochondrial and nuclear markers. We found asymmetric hybridization in a limited zone and tried to explain how the complex geographic history of the region might contribute to the observed genetic pattern.

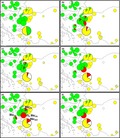

## 1 INTRODUCTION

Climate and habitat changes can facilitate range movements, which can cause secondary contacts between species or lineages (Taylor, Larson, & Harrison, 2015). The Quaternary glacial cycles have strongly influenced the current distributions of Mediterranean species and their genetic diversity (Hewitt, 1999; Schmitt, 2007). Glacial periods occurred ca. every 100,000 years and forced species to contract their ranges into restricted refugia, while interglacial periods allowed them to expand northward (the Expansion‐Contraction model) (Taberlet, Fumagalli, Wust‐Saucy, & Cosson, 1998). In regions strongly affected by glaciations, most of the genetic diversity has accumulated in lower latitudes (Petit et al., 2003), while northern populations show decreased allelic richness and signs of demographic expansions, a pattern known as “southern richness and northern purity” (Hewitt, 1999). Recolonization routes of the species during interglacials were shaped by major biogeographic factors such as barriers and corridors, and by biotic interactions in some cases. The glacial cycles have also affected sea levels and land configurations, thereby modifying landmass connectivity, and thus possible dispersal routes, over time (Hewitt, 2011). The genetic footprints of species' responses to these successions of climate changes have been extensively studied for many species in Europe and North America, while studies in the Near East remain scarce.

**Figure 1:**
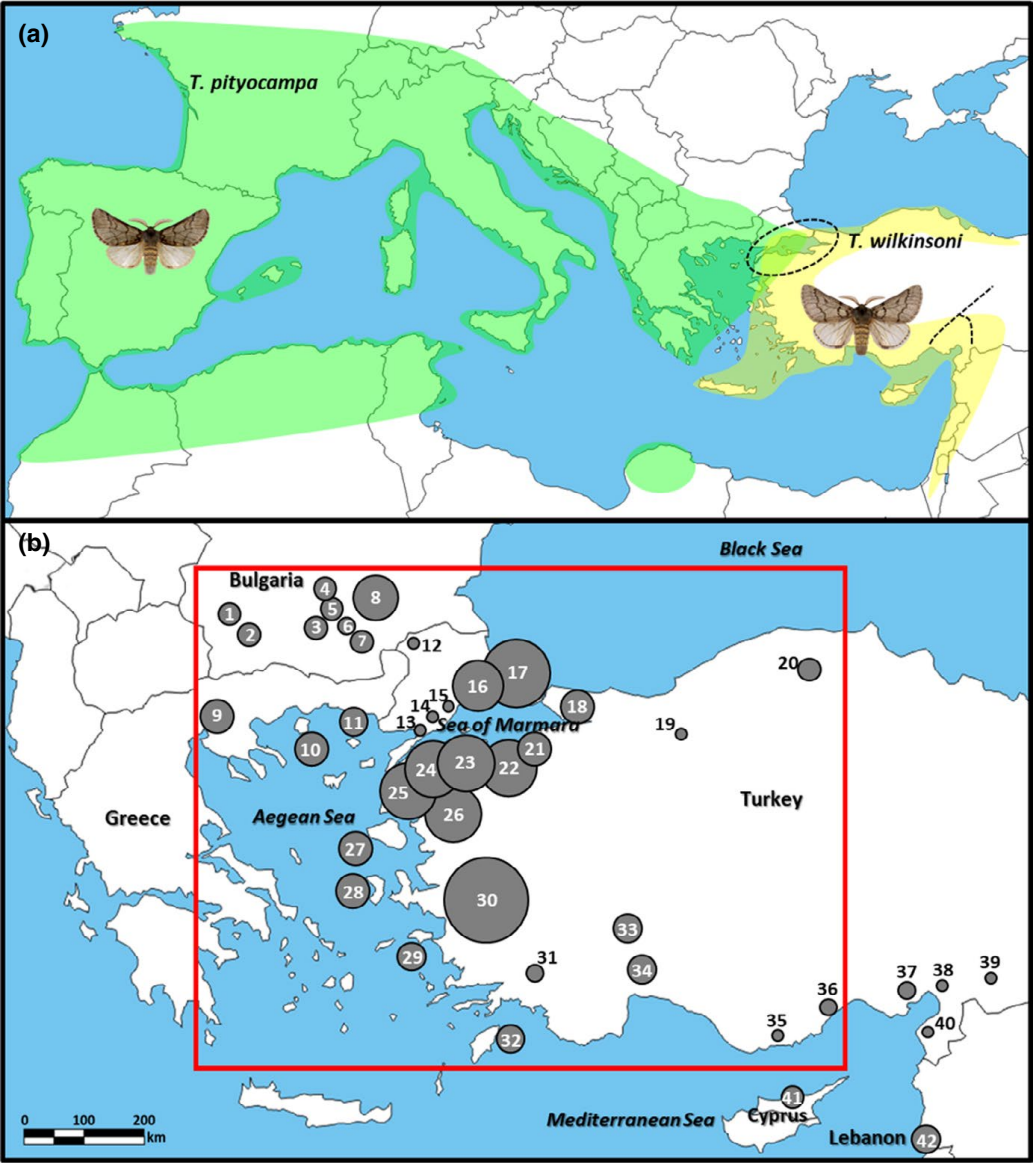
(a) Distribution of the two processionary moth species in the Mediterranean basin (green: *Thaumetopoea pityocampa*; yellow: *T. wilkinsoni*) (dashed circle: Turkish Straits System; dashed line: Anatolian Diagonal) (b) Sampling localities used in the present study. Different circle sizes correspond to different sampling sizes from 2 to 18 individuals. Numbers correspond to the locality codes given in Table 1 (red frame: microsatellite dataset used for hybrid detection analyses, see text for details)

The biogeographic dynamics of the Mediterranean region were affected by two main geologic events: (a) the opening of the Mid‐Aegean Trench (MAT) (~12 Mya), which separated Greek and Turkish peninsulas, and (b) the “Messinian Salinity Crisis” (5.9–5.3 Mya), a major drop in sea level that allowed several landmasses to emerge and connect previously isolated islands (Krijgsman et al., 1999; Poulakakis et al., 2015). The Aegean region is located on a crossroad between continents where contacts between divergent lineages moving from Europe to Anatolia or vice versa are likely (Dubey et al., 2007). Various biogeographic barriers such as the Turkish Straits System (TSS) (Dardanelles—Sea of Marmara—Bosphorus) and the Anatolian Diagonal (Figure 1a), a mountain range extending from southern to northeastern Turkey, make the Eastern Mediterranean a promising area for studying suture zones. Indeed, an increasing number of studies in Turkey have identified secondary contact zones (Bilgin, 2011) and showed that Anatolia was a major glacial refugium for many organisms (e.g., Biltekin et al., 2015; Korkmaz, Lunt, Çıplak, Değerli, & Başıbüyük, 2014; Mutun, 2010). Therefore, biogeographic consequences of the particularly complex geological history and dynamics of landmass configuration in the Aegean region (e.g., Poulakakis et al., 2015) render it a good candidate to study possible past and current hybridization events between lineages or sister species, which is the general frame of the present work.

Most hybrid zones arise from secondary contact in so‐called “Suture Zones” (Hewitt, 1999, 2011), which usually occur at the crossroad between specific dispersal routes and are facilitated by biogeographic discontinuities. They correspond to relatively narrow regions, where gene flow between related taxa leads to recombination of parental species’ alleles or to discordances between nuclear and mitochondrial genomes. This reveals the porous nature of the genome and semipermeability of species boundaries (Harrison & Larson, 2014). An informative way of considering hybrid zones is to determine where they lie in the continuum from uni‐ to bimodality (Harrison & Bogdanowicz, 1997; Jiggins & Mallet, 2000) and to characterize their width, which brings information about the distance reached by introgressed genes (i.e., genes of one species included in the genome of the other species). Unimodal hybrid zones correspond to regions where most individuals have intermediate genotypes compared to the two parental species in contact; these intermediate genotypes have also been called "hybrid swarms" (e.g., Scriber & Ording, 2005). On the other hand, bimodal hybrid zones consist largely of genotypes resembling the parental forms with few intermediates (Jiggins & Mallet, 2000). They usually indicate that speciation of parental forms is nearly complete and is strongly associated with assortative mating or fertilization failure, rather than postzygotic isolation (Jiggins & Mallet, 2000). Both uni‐ and bimodality can occur when two allopatric sister species come in secondary contact, or at the border of the distributions of in situ parapatric species.

The winter pine processionary moth (PPM) (Lepidoptera: Notodontidae) comprises two univoltine allopatric Mediterranean sister species (Basso, Negrisolo, Zilli, Battisti, & Cerretti, 2017; Kerdelhué et al., 2009), *Thaumetopoea pityocampa* (Denis & Schiffermüller, 1775) in the western Mediterranean (from Portugal to western Turkey and in North Africa), and *Thaumetopoea wilkinsoni* Tams, 1925, in the Eastern Mediterranean. Divergence of their mitochondrial genomes was estimated to date back to the late Miocene (Kerdelhué et al., 2009). Several glacial refugia were identified for *T. pityocampa*, mostly in the south of the Mediterranean Basin and southern Europe (Kerdelhué et al., 2009; Rousselet et al., 2010). A major postglacial demographic expansion occurred throughout Europe after the last glacial maximum, but its history in the eastern part of its range remains poorly documented, except in Greece (Korsch et al., 2015). Previous studies revealed four differentiated mitochondrial lineages within *T. wilkinsoni*: Crete, Cyprus, western Turkey, and eastern Turkey–Israel (Kerdelhué et al., 2009; Simonato et al., 2007) (the population in Crete might be a separate species, see Petsopoulos et al., 2018). However, biogeographic patterns of *T. wilkinsoni* in Turkey and western limits of its distribution have been largely unexplored.

A contact zone between *T. pityocampa* and *T. wilkinsoni* was recently discovered in western Turkey with some evidence of introgression (İpekdal, Burban, Kerdelhué, & Çağlar, 2015). However, this result was based on sparse sampling and a limited number of mitochondrial and nuclear markers. Petrucco‐Toffolo et al. (2018) further demonstrated the viability and fecundity of F_1_ individuals without any sign of outbreeding depression in no‐choice laboratory hybridization experiments. Apart from these two studies, there is no other study on PPM hybridization, details of which, therefore, have remained unknown so far.

The objectives of the present study were (a) to infer their local phylogeographic history and delineate their co‐occurrence patterns, (b) to identify natural hybrids and explore the genetic legacy of interspecific gene flow between the two species, and (c) to determine how the biogeographic context of the Aegean region influenced species range shifts and formation of a hybrid zone.

## 2 MATERIALS AND METHODS

### 2.1 Sampling

We sampled 174 larvae from 27 localities in Greece, Bulgaria, Turkey, Cyprus, and Lebanon (major regions related to the suspected hybrid zone and regions that were ignored or insufficiently studied previously) between 2002 and 2012. Fifty specimens from İpekdal et al. (2015) were also included; thus, we obtained 224 individuals from 42 localities in total (Figure 1; Table 1). Larvae (first–fifth instars) were collected from different host trees to avoid sampling siblings and stored at −20°C in 70% ethanol.

**Table 1:** Geographic locations, sampling dates, host tree species, sample size (*n*), mitochondrial COI haplotypes, and nuclear Pho alleles. Codes for COI haplotypes and Pho alleles are the same as in Figure 2

Code	Sampling locality	Coordinates		Date	Host tree	*n*	mt COI Haplotype	nuc Pho Allele
1	Sandanski, Bulgaria	41°34′	23°17′	2004/02/12	*P. nigra*	4	1	1, 2
2	Marikostinovo, Bulgaria	a	—	2005/09/05	*—*	4	1, 2	1, 2
3	Plovdiv, Bulgaria	—	—	2003/05/22	*—*	4	1	1, 2
4	Banya, Bulgaria	42°33′	24°50′	2005/08/18	*—*	4	1	1
5	Javrovo, Bulgaria	41°59′	24°47′	2005/06/27	*P. nigra*	4	4, 5	2
6	Asenovgrad, Bulgaria	—	—	—	*—*	3	1	1, 2
7	Zvezdel, Bulgaria	—	—	—	*—*	4	1, 9, 10	1, 2
8	Haskovo Bulgaria	—	—	—	*—*	8	1, 3	2
9	Thessaloniki, Greece	40°28′	23°23′	2002/08/30	*P. brutia*	6	1, 8	1, 2
10	Thasos, Greece	40°46′	24°42′	—	*P. halepensis*	6	1	1, 2
11	Samothraki, Greece	40°29′	25°31′	—	*P. halepensis*	5	1	1, 2
12	Edirne, European Turkey	41°23′	26°48′	2008/12/20	*P. nigra*	2	1	2
13	Gelibolu, European Turkey	40°34′	26°49′	2008/12/20	*P. nigra*	2	1	2
14	Korudağı, European Turkey	40°44′	26°43′	2008/12/20	*P. nigra*	2	1	2
15	Gölcük, European Turkey	40°40′	27°04′	2008/12/20	*P. nigra*	2	1	2, 8
16	Tekirdağ, European Turkey	40°50′	27°26′	2011/01/26	*P. nigra*	9	1, 16	2, 8
17	İstanbul, European Turkey	41°06′	29°01′	2011/01/26	*P. nigra*	12	14	2, 8
18	İzmit, Northern Turkey	40°48′	29°57′	2011/01/26	*P. nigra*	6	16	8
19	Bolu, Northern Turkey	40°45′	31°34′	2008/12/18	*P. nigra*	2	14	8
20	Kastamonu, Northern Turkey	41°21′	33°45′	2010/04/29	*P. brutia*	4	24	8
21	Mudanya, Southern Marmara	40°23′	28°49′	2011/01/16	*P. brutia*	6	14, 19	8
22	Karacabey, Southern Marmara	40°12′	28°21′	2011/01/16	*P. brutia*	10	20	8
23	Biga, Southern Marmara	40°14′	27°16′	2011/01/17	*P. brutia*	10	1	1, 2, 8
24	Çanakkale, Southern Marmara	40°05′	26°23′	2011/01/17	*P. brutia*	10	1, 6, 11	1, 2, 3
25	Ezine, Southern Marmara	39°42′	26°22′	2011/01/18	*P. brutia*	10	11, 12, 13	1, 2, 8
26	Burhaniye, Aegean Turkey	39°30′	26°57′	2011/01/18	*P. brutia*	10	1	1, 2, 8
27	Lesvos, Greece	39°10′	25°56′	—	*P. halepensis*	6	1, 7	1, 2, 8
28	Chios, Greece	38°22′	26°01′	—	*P. halepensis*	6	1	1, 2, 8
29	Samos, Greece	37°42′	27°00′	—	*P. brutia*	5	14, 17, 21, 26	8
30	İzmir, Aegean Turkey	38°34′	27°20′	2008/09/01	*P. brutia*	18	1, 14, 18, 22, 23	1, 4, 8, 10
31	Muğla, Aegean Turkey	36°46′	28°50′	2008/09/03	*P. brutia*	1	27	8
32	Rhodes, Greece	36°24′	28°08′	—	*P. brutia*	5	14, 15, 17, 25, 28	8, 9
33	Burdur, Southern Turkey	37°38′	30°39′	2008/09/04	*P. brutia*	5	29, 30, 31, 32, 33	8
34	Antalya, Southern Turkey	36°56′	30°53′	2010/03/09	*P. brutia*	5	34, 35, 36, 37	8
35	Anamur, Southern Turkey	36°02′	32°47′	2010/03/09	*P. brutia*	2	38, 39	8
36	Silifke, Southern Turkey	36°18′	33°51′	2010/03/10	*P. brutia*	3	40, 41, 42	8
37	Tarsus, Southeastern Turkey	36°56′	34°49′	2010/03/10	*P. brutia*	3	44, 45	5, 8
38	İskenderun, Southeastern Turkey	36°32′	36°09′	2010/03/10	*P. brutia*	2	43, 47	8
39	Kilis, Southeastern Turkey	36°48′	36°58′	2010/03/10	*P. brutia*	2	46	7, 8
40	Hatay, Southeastern Turkey	36°08′	36°04′	2010/03/11	*P. brutia*	2	48	7, 8
41	Kyrineia, Cyprus	35°19′	33°35′	2008/10/23	*P. brutia*	4	50, 51	5, 6
42	Beirut, Lebanon	—	—	—	*—*	5	49	5, 8

Abbreviation: NA, not available.

a missing data due to incomplete collector records.

### 2.2 Laboratory protocols

We extracted larval genomic DNA (full body or head) using DNeasy tissue kit (Qiagen) and sequenced the samples for an 810 bp fragment of the mitochondrial Cytochrome c Oxidase subunit I gene (COI) using the primers Jerry/Pat (Rousselet et al., 2010), and a 660 bp fragment of the nuclear Photolyase gene (Pho) using the primers from Simonato et al. (2013). MWG Company performed sequencing on an ABI PRISM 3730 genetic analyzer using BigDye Terminator chemistry (Applied Biosystems). Whenever we obtained heterozygous sequences (16 individuals) for Pho, we cloned PCR products using pGEM T Easy Vector (Promega Corp.). We sequenced six to eight clones per individual to obtain the phased sequence of both alleles. Additionally, we characterized the Internal Transcribed Spacer 1 (ITS‐1) by RFLP‐PCR for all 224 individuals. We amplified the ITS‐1 fragment using the primers from Vogler and DeSalle (1994) and digested it using the restriction enzyme Hga‐1, which produces different banding patterns for *T. pityocampa* (6 bands of 152, 131, 97, 70, 33, and 17 bp based on the GenBank accession EF189684), and *T. wilkinsoni* (5 bands of 232, 153, 66, 33, and 17 bp, accession EF189687). To check the consistency of results, we cloned and sequenced 13 individuals showing typical banding pattern for the species and 5 individuals with an ambiguous or hybrid‐like banding pattern. We obtained high‐quality consensus sequences using CodonCode Aligner 1.63 and aligned using ClustalW 4.0 (Larkin et al., 2007). We checked sequences for stop codons and double peaks to avoid pseudogenes. We also used 12 microsatellite markers (MS‐Thpit‐01, MS‐Thpit‐03, MS‐Thpit‐04, MS‐Thpit‐05, MS‐Thpit‐08, MS‐Thpit‐11, MS‐Thpit‐12, MS‐Thpit‐13, MS‐Thpit‐15, MS‐Thpit‐16, MS‐Thpit‐18, MS‐Thpit‐19) (A'Hara et al., 2012; Rousselet, Magnoux, & Kerdelhué, 2004). We performed genotyping with an ABI‐3730 automatic sequencer at the Plateforme Génome Transcriptome (Bordeaux) and conducted allele sizing with GeneMapper 4.0 (Applied Biosystems).

### 2.3 Analyses of sequence and RFLP data

We built COI haplotype and Pho allelic networks using TCS 1.21 (Clement, Posada, & Crandall, 2000), including *T. pityocampa* and *T. wilkinsoni* reference sequences downloaded from GenBank (COI: GU385906, EF185140; Pho: JX182493, JX182496). We then calculated the number of haplotypes and alleles using DnaSP‐5.10.01 (Librado & Rozas, 2009). Genetic *p*‐distances between any COI haplotype (respectively Pho alleles) found in the present study and the reference sequence downloaded from GenBank were further calculated using MEGA 6 (Tamura, Stecher, Peterson, Filipski, & Kumar, 2013). We analyzed between and within‐group distances for COI haplotypes assigning lineages and species as groups and using Kimura 2‐parameter (Kimura, 1980) in MEGA X (Kumar, Stecher, Li, Knyaz, & Tamura, 2018).

By examining the respective networks and corresponding *p*‐distances, each haplotype/allele could be considered as characteristic of one or the other species. Taking also into account their ITS RFLP patterns, hybrid individuals were identified based on two criteria: (a) the co‐occurrence of allele's characteristic of both species for at least one nuclear marker, or (b) the incongruence (mt‐nuc or nuc‐nuc) between the specific diagnosis of the different markers.

### 2.4 Analyses of microsatellite data

#### 2.4.1 Population genetic structure

We performed a Principal Component Analysis (PCA) using the adegenet 1.4‐2 package (Jombart, 2008) implemented in R (R Core Team, 2018). We assigned individuals to clusters according to their membership coefficients (*q*) using a Bayesian inference method implemented in STRUCTURE 2.3 (Pritchard, Stephens, & Donnelly, 2000) with Admixture Model and correlative allelic frequency option. We used 100,000 burn‐in cycles followed by 100,000 MCMC simulations and performed 10 iterations for each *K* (number of clusters) from 1 to 6. We selected the *K* value that best fit the data by examining the curve of ln P(X|*K*) and calculating Δ*K* (Evanno, Regnaut, & Goudet, 2005). After checking consistency of results over all iterations, we used the greedy algorithm of CLUMPP 1.1.2 (Jakobsson & Rosenberg, 2007) to average results of all the runs for each K. Results were plotted using DISTRUCT 1.1 (Rosenberg, 2004).

#### 2.4.2 Hybrid detection from microsatellites

To identify each individual as pure *T. pityocampa*, pure *T. wilkinsoni,* or hybrid (F_1_, F_2_, or backcross) from their microsatellite genotypes, we followed the strategy developed in Burban et al. (2016) using the field dataset together with simulated genotypes of known ancestry (see below). Briefly, identification of hybrid individuals is based on complementary methods whose respective performance is dependent both on the power of the data set available (type and number of markers, sampling size) and of the level of differentiation and reproductive isolation. Therefore, using a combination of methods and a case‐specific evaluation of their performances through analyses of simulated genotypes is highly recommended (Marie, Bernatchez, & Garant, 2011; Vähä & Primmer, 2006). STRUCTURE is first used to delimit the clusters that are expected to hybridize (parental clusters). This first step then allows to infer the proportion of alleles inherited from each parental species trough *h*‐index estimation using INTROGRESS (Buerkle, 2005) and is also useful to generate simulated genotypes of known ancestry. A complementary approach is provided by NewHybrids (Anderson & Thompson, 2002) that potentially discriminates specifically first (F_1_) and second (F_2_ and backcrosses) hybrid generations from parental individuals. Note that based on the first STRUCTURE analyses and PCA, we excluded individuals from southeastern Turkey, Cyprus, and Lebanon (localities 37–42) to develop this procedure of hybrid identification, because these sites are distant from the potential contact zone and correspond to genetically differentiated lineages of *T. wilkinsoni*.

##### Simulated dataset

We selected all individuals having STRUCTURE *q‐*value (hereafter, *q*_STR_) ≥0.900 in their respective cluster at *K* = 2 (102 *T. pityocampa* and 98 *T. wilkinsoni* individuals) to generate a simulated dataset consisting of 1,000 *T. pityocampa* (sim*_Pit_*), 1,000 *T. wilkinsoni* (sim*_Wil_*), and 4,000 hybrid individuals (1,000 for each of the first‐ and second‐generation hybrids [F_1_ and F_2_], backcrosses with parental *T. pityocampa* [Bkc*_Pit_*] and *T. wilkinsoni* [Bkc*_Wil_*]) using HYBRIDLAB (Nielsen, Bach, & Kotlick, 2006). For each simulated category, we estimated the range of assignment indices obtained with STRUCTURE, INTROGRESS, and NewHybrids to support the interpretation of the field dataset.

##### STRUCTURE

We analyzed the field dataset together with the 6,000 simulated individuals and determined the range of individual q‐values (*q*_STR_) corresponding to sim*_Pit_* and sim*_Wil_*. Any field individual having a *q*_STR_ value outside this range was considered as “nonparental,” that is, hybrid.

##### INTROGRESS

We calculated the hybrid index (*h*‐index) using the est.h function of the INTROGRESS package (Gompert & Buerkle, 2010). We used sim*_Pit_* and sim*_Wil_* as reference parental individuals to calculate *h*‐index for field and simulated individuals, the values ranging from 0 (pure *T. pityocampa* ancestry) to 1 (pure *T. wilkinsoni* ancestry). Finally, hybrids were identified among the field individuals when their *h*‐index fell out of the range of simulated parents.

##### NewHybrids

This Bayesian software estimates posterior probabilities (*q*_NH_) of each individual to fall into one of the six genotypic classes described above. We first ran NewHybrids on the simulated dataset alone using Jeffreys‐like priors for allele frequencies and mixing proportions. We used 5 iterations of this analysis to check consistency of results and obtain averaged *q*_NH_ values across runs. We used the “majority assignment” method to assign each individual to the class corresponding to the highest *q*_NH_, as described in Burban et al. (2016). We compared the known genetic class of each simulated individual to the corresponding assignment result and used Vähä and Primmer's (2006) measures of efficiency, accuracy, and overall performance to estimate the power of assignment of the method with our dataset. Calculations of these measures are as follows:

$$\text{efficiency}={\text{number of simulated F}}_{1}{\;\text{correctly assigned to F}}_{1}\;\text{class for the given}\;q/{\text{number of individuals in the simulated F}}_{1}\;\text{class}$$

$$\text{accuracy}={\text{number of simulated F}}_{1}{\;\text{correctly assigned to F}}_{1}\;\text{class for the given}\;q/{\text{number of individuals assigned to F}}_{1}\;\text{class either correctly or incorrectly for the given}\;q$$

overall performance=efficiency/accuracy

Additionally, to characterize the potential resulting errors, we examined the type of misassignments obtained for each class of simulated genotype. We then ran NewHybrids as described above, using the field and simulated individuals together to avoid any bias due to unbalanced occurrence of each genotypic class.

## 3 RESULTS

### 3.1 Mitochondrial and nuclear sequences and RFLP data

#### COI

**Figure 2:**
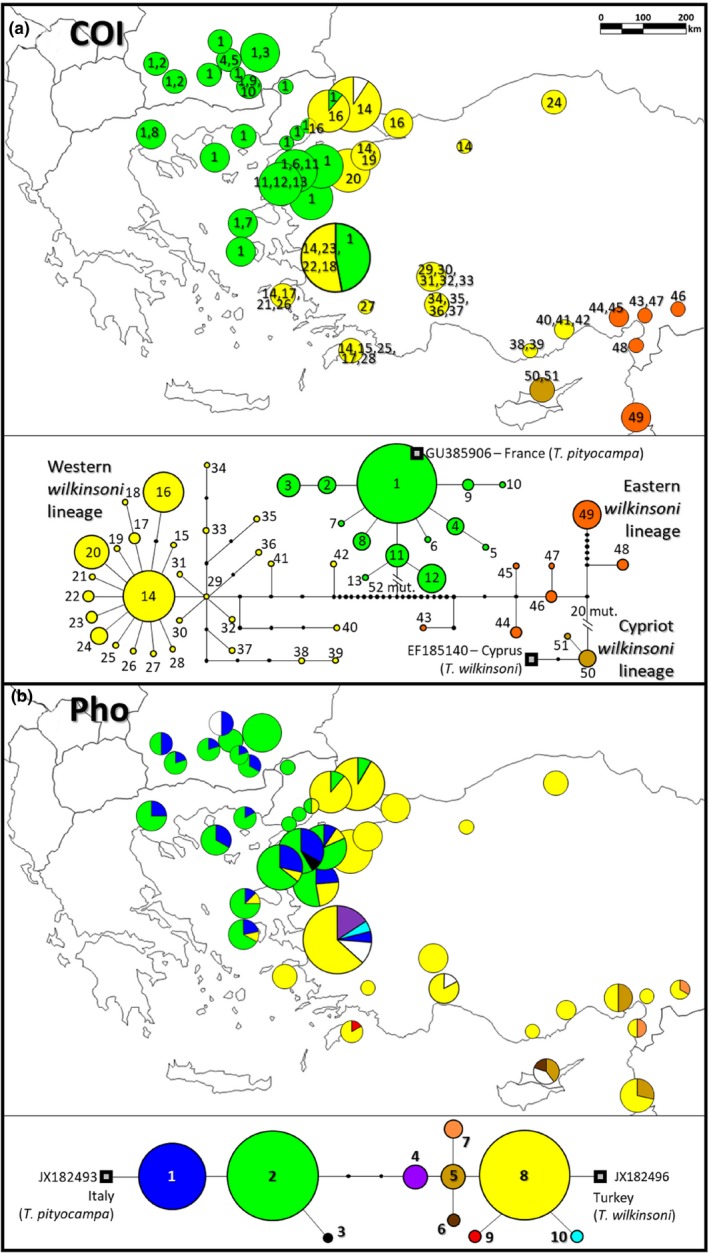
Networks and geographic distribution of (a) COI haplotypes 1–13 [green]: *T. pityocampa*; 14–51: *T. wilkinsoni* (14–42 [yellow]: western *wilkinsoni* lineage; 43–49 [orange]: eastern *wilkinsoni* lineage; 50–51 [brown]: Cypriot *wilkinsoni* lineage), (b) Pho alleles 1–3: *T. pityocampa*, 4–10: *T. wilkinsoni*. White color in the pies corresponds to missing data

We found 51 COI haplotypes that corresponded to two distinct clades (between‐group mean distance: 0.0971). When compared to the reference sequences, these two clades corresponded to the two species, with 13 haplotypes and 121 individuals for *T. pityocampa* (within‐group mean distance: 0.0032) and 38 haplotypes and 102 individuals for *T. wilkinsoni* (within‐group mean distance: 0.0195) (Figure 2a). The *pityocampa* clade had a star‐like pattern, while *wilkinsoni* included three subclades, the mean distance between subclades being comprised between 0.0292 and 0.0332 (1—Cyprus, the Cypriot *wilkinsoni* lineage; 2—southeastern Turkey and Lebanon, hereafter the eastern *wilkinsoni* lineage [localities 37–40 and 42]; 3—Europe, western Turkey and adjacent islands, hereafter the western *wilkinsoni* lineage) as expected from previous phylogenetic analyses (Kerdelhué et al., 2009; Simonato et al., 2007) (Figure 2a). The southernmost Turkish populations (31, 33–36) hosted diversified and rare haplotypes, while all other haplotypes formed a star‐like network. Individuals either with *T. pityocampa* or *T. wilkinsoni* haplotypes co‐occurred in localities Gölcük‐15, Tekirdağ‐16, and İzmir‐30.

#### Pho

10 Pho alleles were found among 217 successfully sequenced individuals. Three alleles were closer to the *pityocampa* reference (alleles 1–3) and corresponded to individuals from Europe and western Turkey, while 7 alleles were closer to the *wilkinsoni* reference (alleles 4–10) and occurred in Turkey, Lebanon, and Cyprus. From this marker, 104 individuals were identified as *T. pityocampa*, 104 as *T. wilkinsoni*, and 9 individuals had alleles from both species. These hybrids were found in Biga (23), Ezine (25), Burhaniye (26), Lesvos (27), Chios (28), and İzmir (30) (Table 2). Moreover, according to this marker, both species occurred in Gölcük (15), Tekirdağ (16), and İstanbul (17) (Figure 2b).

**Table 2:** Genetic characteristic of individuals considered as hybrid either from discordance between species assignment of the Pho and ITS alleles and COI haplotypes (p: *pityocampa*, w: *wilkinsoni*), or from one of the hybrid detection methods based on microsatellite data (STR: STRUCTURE, NH: NewHybrids, INT: INTROGRESS). Scores in STR column correspond to *q*‐values for the *wilkinsoni* cluster, whereas those in INT column correspond to *h*‐indices (0 = pure *pityocampa*, 1 = pure *wilkinsoni*). Majority assignment is given for NH. *f*_H_ is the frequency of inferred hybrids in the corresponding sampling

Locality No	Locality	COI	Pho	ITS−1	μsat			*f*_H_
					STR	NH	INT	
15	Gölcük	p	p	w,p	p (0.072)	p	p (0.044)	0.50
16	Tekirdağ	p	p	w	p (0.118)	p	p (0.077)	0.11
23	Biga	p	w,p	p	p (0.057)	p	p (0.012)	0.40
		p	p	p	p (0.145)	Bkc*_Pit_*	p (0.109)	
		p	p	p	p (0.176)	Bkc*_Pit_*	p (0.141)	
		p	p	w,p	p (0.063)	p	p (0.047)	
25	Ezine	p	w,p	p	p (0.092)	p	p (0.000)	0.10
26	Burhaniye	p	w,p	p	0.422	F_2_	0.413	
		p	p	p	0.395	F_2_	0.385	
		p	w,p	p	0.416	F_2_	0.452	
		p	p	p	p (0.204)	Bkc*_Pit_*	p (0.146)	
		p	p	w,p	0.298	Bkc*_Pit_*	0.276	1.00
		p	w,p	p	p (0.200)	Bkc*_Pit_*	p (0.179)	
		p	p	p	p (0.138)	Bkc*_Pit_*	p (0.103)	
		p	w,p	p	0.455	F_2_	0.443	
		p	p	p	0.466	F_2_	0.450	
		p	p	p	0.342	F_2_	0.326	
27	Lesvos	p	p	p	0.209	Bkc*_Pit_*	p (0.069)	
		p	w,p	p	p (0.042)	p	p (0.176)	0.50
		p	p	p	p (0.195)	Bkc*_Pit_*	0.198	
28	Chios	p	w,p	p	p (0.052)	p	p (0.000)	0.17
30	İzmir	p	w	w	w (0.956)	w	w (1.00)	0.56
		p	w	—	—	—	—	
		p	w	w	0.736	Bkc*_Wil_*	0.757	
		w	w	w	0.806	Bkc*_Wil_*	0.834	
		p	w	w	w (0.960)	w	w (1.00)	
		p	w	w	w (0.924)	w	w (0.954)	
		p	w	w	w (0.925)	w	w (0.957)	
		p	w	w	w (0.859)	w	w (0.890)	
		p	w,p	w,p	w (0.896)	w	w (0.923)	
		w	w	w	0.744	Bkc*_Wil_*	0.764	

#### ITS‐1

We obtained ITS‐1 RFLP data for 213 out of 224 individuals, corresponding to 107 *pityocampa* and 102 *wilkinsoni* patterns as well as 4 hybrids found in Gölcük (15), Biga (23), Burhaniye (26), and İzmir (30) (Table 2). These results were confirmed by cloning and sequencing these 4 individuals along with 3 *pityocampa* and 5 *wilkinsoni* reference individuals. Only one locality (İstanbul, 17) contained both species according to this marker.

Apart from individuals that had alleles from two different species for at least one marker, we also found individuals having “taxonomical discordance” between their mitochondrial and nuclear sequences and/or microsatellite markers (mt‐nuc), or among the nuclear sequences (nuc‐nuc) studied (Table 2). All the individuals having mt‐nuc discordance had a *pityocampa* mitochondrial haplotype (Table 2). Geographic distributions of the two species and their hybrids according to each marker are shown in Figure 5.

### 3.2 Microsatellite data

#### 3.2.1 Population genetic structure

##### PCA

**Figure 3:**
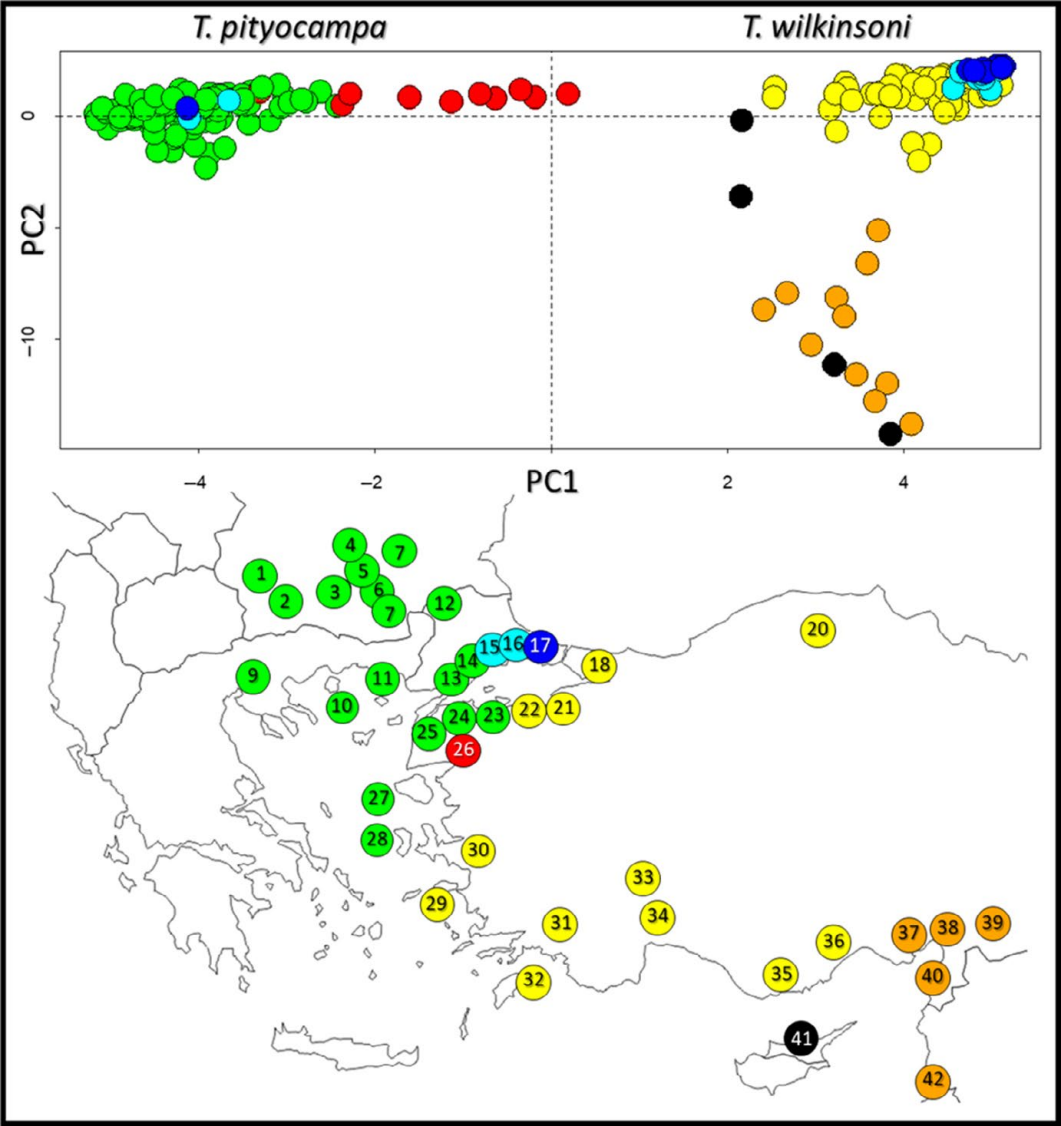
Plot of PCA scores based on the complete microsatellite data and geographic distribution of populations color‐coded according to the PCA plot. Green: main *T. pityocampa* populations, yellow: main *T. wilkinsoni* populations, orange: *T. wilkinsoni* populations in southeastern Turkey, black: *T. wilkinsoni* populations in Cyprus, dark blue: *T. pityocampa* and *T. wilkinsoni* populations occurring at the same locality without any trace of hybridization found, light blue: *T. pityocampa* and *T. wilkinsoni* populations occurring at the same locality with some traces of hybridization, red: population 26 with individuals positioned more centrally along the PC1

The first two axes of the PCA explained 7.5% and 3.4% of the total inertia, respectively. PC1 separated the two species. Three populations (Gölcük, 15; Tekirdağ, 16; İstanbul, 17) contained individuals from the two groups, while most individuals from Burhaniye (26) exhibited intermediate positions along PC1. PC2 separated southeastern Turkey (37–40), Cyprus (41), and Lebanon (42) from rest of Turkey within the *wilkinsoni* clade (Figure 3).

##### STRUCTURE

**Figure 4:**
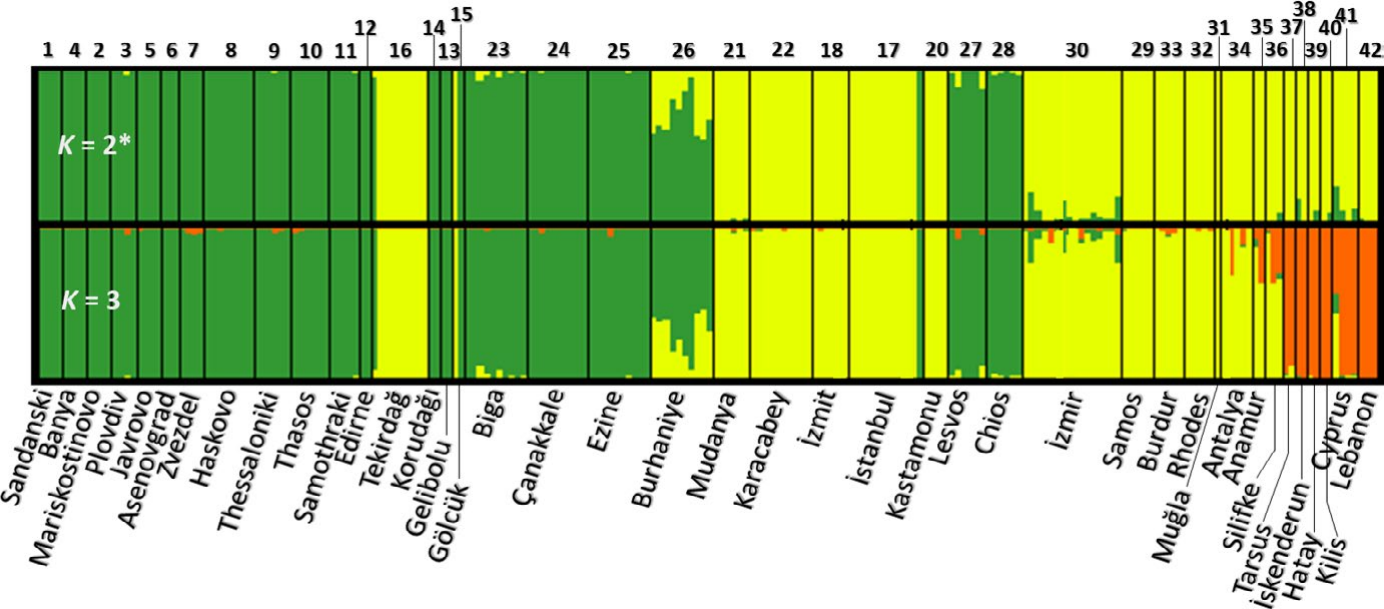
Individual clustering for *K* = 2 and *K* = 3 based on STRUCTURE analysis of microsatellite data from the complete field dataset. Numbers above and names below are codes and names of the sampling localities, respectively

*K* = 2 was the optimal number of clusters identified both with lnP(X|*K*) and Δ*K* methods. Most individuals were clearly assigned to one or the other cluster, that is, to either *T. pityocampa* or *T. wilkinsoni* (Figure 4). Consistently with the PCA results, three populations (Gölcük, 15; Tekirdağ, 16; İstanbul, 17) included both species. On the other hand, most individuals from Burhaniye (26) had relatively low assignment scores. At *K* = 3, southeastern Turkey, Cyprus, and Lebanon formed a separate cluster.

#### 3.2.2 Hybrid detection from microsatellite data

##### STRUCTURE

From the analysis of the simulated data, we found that pure parental *T. pityocampa* and *T. wilkinsoni* individuals had *q*‐values below 0.207 and above 0.809, respectively. Applying these thresholds to the field individuals, 11 individuals were assigned as hybrids (Table 2).

##### INTROGRESS

The *h*‐index ranged from 0.000–0.183 for sim*_Pit_* and 0.838–1.000 for sim*_Wil_*. When applying these ranges for the field individuals analyzed together with the simulated individuals, 105 individuals were assigned as *T. pityocampa*, and 85 as *T. wilkinsoni*. Eleven individuals had an *h*‐index outside of this range and were thus considered as hybrids.

##### NewHybrids

Efficiencies, accuracies, and overall performances were generally high for the simulated dataset, except for F_2_ (Table 3). The levels of misassignment for each class of simulated individuals are shown in Table 3. Among field individuals, 17 were assigned as hybrid (6 F_2_, 8 Bck*_Pit_*, and 3 Bck*_Wil_*, Table 2).

**Table 3:** Efficiency, accuracy, overall performance of NewHybrids to characterize parental and hybrid categories from simulated genotypes (lines) and their proportion to each category (columns) by using the majority assignment approach

	Efficiency	Accuracy	Performance	P*_Pit_*	P*_Wil_*	F_1_	F_2_	Bkc*_Pit_*	Bkc*_Wil_*
sim*_Pit_*	0.983	0.957	0.941	0.983	0	0	0	0.017	0
sim*_Wil_*	0.998	0.983	0.981	0	0.998	0	0	0	0.002
simF_1_	0.978	0.921	0.901	0	0	0.978	0.006	0.006	0.010
simF_2_	0.767	0.898	0.689	0.001	0	0.043	0.767	0.110	0.079
simBkc*_Pit_*	0.902	0.871	0.786	0.043	0	0.012	0.043	0.902	0
simBkc*_Wil_*	0.916	0.909	0.833	0	0.018	0.028	0.038	0	0.916

To sum up, we found a high but not strict consistency of results between the three methods. The 11 individuals identified as hybrids from INTROGRESS were also identified as such by NewHybrids, and 10 of them by STRUCTURE (Table 2). However, the methods used did not allow to test more complex ancestries, such as recurrent backcrosses.

**Figure 5:**
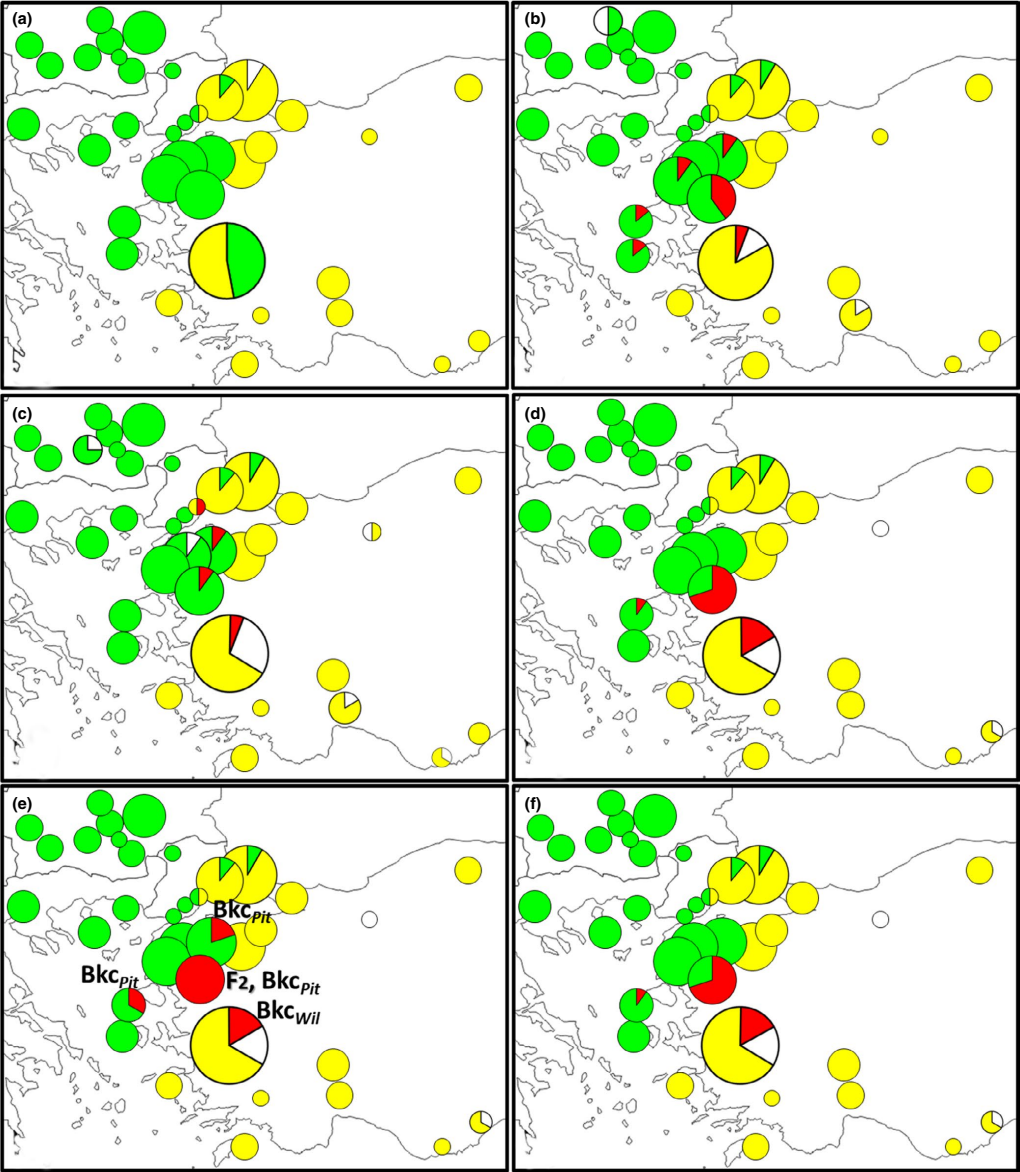
Distribution of *T. pityocampa* (green), *T. wilkinsoni* (yellow), and their hybrids (red) (white: missing data) according to (a) COI, (b) Pho, (c) ITS‐1, (d) STRUCTURE, (e) NewHybrids (F_2_: second‐generation hybrid, Bkc*_Pit_*: backcross with *T. pityocampa*; Bkc*_Wil_*: backcross with *T. wilkinsoni*), and (f) INTROGRESS analyses (see Table 2 for details)

All individuals considered as hybrid from these analyses were collected in a restricted region comprising the Turkish Strait System (Table 2). Moreover, hybrid individuals having scores closer to pure *T. pityocampa* (<0.5 for STRUCTURE and INTROGRESS, Bck*_Pit_* for NewHybrids) were found to be geographically closer to the *T. pityocampa* range in the western part of the studied region, while hybrids genetically closer to pure *T. wilkinsoni* (>0.5 for STRUCTURE and INTROGRESS, Bck*_Wil_* for NewHybrids) were located near the *T. wilkinsoni* range in the eastern part of the zone (Figure 5e).

## 4 DISCUSSION

A large majority of the samples were consistently assigned to one of the species across all markers (86% of the studied individuals), which allows to document the parapatric distribution and contrast phylogeographic patterns between the two species (discussed below). Pure *T. wilkinsoni* was present in Lebanon, Cyprus, and in most Turkish sites (including the Bosphorus and the Thrace regions) as well as in the Greek islands of Samos in the north and Rhodes in the south, but, remarkably, it was absent from the Turkish Dardanelles (Figure 2). Pure *T. pityocampa* occurred in Bulgaria, mainland Greece, Thasos, and Samothrace islands in northeastern Aegean Sea, and the western side of the Bosphorus in Turkey. It also noticeably occurred on both sides of the Turkish Dardanelles, and as south as Lesvos and Chios, Greek islands close to the Turkish mainland. From a biogeographic perspective, faunas in the Aegean islands are expected to be similar to those of the nearest mainland as a consequence of either Pleistocene land connections during glacial periods (Poulakakis et al., 2015) or, simply, current genetic exchange facilitated by the short distance between the island and the continent (Kerdelhué et al., 2009). This is true for most of the islands included in the present study except for Chios where we found *T. pityocampa*, whereas *T. wilkinsoni* was on the nearest Turkish coast. Moreover, all the hybrid individuals found (14%; note that no hybrid F_1_ were identified) were only in eight sampling sites restricted to western Turkey, delimiting a relatively narrow contact zone where the two species can hybridize.

### 4.1 Contrasting phylogeographic patterns of the two sister species

Consistent with previous studies, we showed that *T. wilkinsoni* has three highly differentiated mitochondrial lineages (Cypriot, eastern, and western *wilkinsoni*), suggesting a long‐lasting presence in isolated refugia. The use of complementary molecular markers and a widespread sampling confirmed this diversification pattern, as mitochondrial and nuclear results were mostly consistent. The strong mitochondrial differentiation of Cypriot *wilkinsoni* lineage, as in Simonato et al. (2007), is probably due to a founder effect and local drift of the maternal lineage (Kerdelhué et al., 2009) and was not accompanied by a strong nuclear divergence, as Cypriot populations grouped predominantly with eastern populations in microsatellite analyses. Such an inconsistency between mitochondrial and nuclear markers was already observed for a mitochondrial clade of *T. pityocampa* in North Africa that was not confirmed by nuclear markers, possibly due to a genetic legacy of an old differentiation of the maternal lineage erased by extensive male gene flow (El Mokhefi et al., 2016). Mountain ridges are well‐known ecological barriers impeding dispersal of species (Schmitt, 2007). Accordingly, our results suggest that the Anatolian Diagonal (Figure 1a), the north‐eastern extension of the Taurus chain, has acted as a major geographic barrier separating the eastern and the western *wilkinsoni* lineages (Figure 2), as is documented for many taxa in Turkey (Gür, 2016). Within the western *wilkinsoni* lineage, the haplotype network revealed two different patterns: a star‐like and a branched pattern. The haplotypes found in western and northern Turkey corresponded to a star‐like pattern, consistent with a typical range expansion phenomenon from southern refugia (possibly including the islands) to the north (Avise, 2000; Slatkin & Hudson, 1991), which suggests a recent postglacial recolonization of these territories. In contrast, numerous endemic haplotypes showing a branched haplotype pattern were detected at low frequency in the Taurus chain, which suggests local survival of populations in these mountains. We also identified a contact zone between the eastern and the western *wilkinsoni* lineages (Anamur, 35; Silifke, 36) (see STRUCTURE *K* = 3 results on Figure 4), possibly caused by recurrent male gene flow from the Adana plain in the east. These results are consistent with the findings of Rousselet et al. (2010), who suggested that mountains of moderate altitudes could serve as refugia rather than barriers to dispersal in the PPM. This pattern recalls the network found for *Tomicus piniperda* (L.) (Coleoptera: Scolytidae) in Europe (Horn, Stauffer, Lieutier, & Kerdelhué, 2009), in which haplotypes from the Iberian Peninsula (a region including multiple local refugia) were diverse and spatially restricted, while haplotypes present elsewhere in Europe formed a star‐like network. Following Bilgin (2011), we suggest that Anatolia should be considered as a collection of multiple small refugia (refugia within refugia, see Gómez & Lunt, 2006) rather than a single refugium. Our results exemplify how the diverse geography of Anatolia has influenced the response of *T. wilkinsoni* to Quaternary glacial cycles, with strong barriers (the Anatolian Diagonal), a diversity of southern local refugia (the Taurus Mountains), and genetically depauperate northern sites.

The recolonization by *T. wilkinsoni* of the northwestern part of its distribution was probably impeded near the Bosphorus by the presence of its sister species *T. pityocampa*. We found one single group of haplotypes for *T. pityocampa*, with a clear star‐like network. This suggests that northeastern Aegean region would not be a long‐lasting refugium for *T. pityocampa* but the edge of its expanded range. The main haplotype found here (haplotype‐1, see Figure 2) is also the major one found all along from eastern France to Greece (Korsch et al., 2015; Rousselet et al., 2010). This confirms the extensive eastward postglacial range expansion of *T. pityocampa* throughout Europe. Previous studies showed that divergent lineages occur for this species in the Iberian Peninsula, Corsica and North Africa (Kerdelhué et al., 2009; Rousselet et al., 2010). Our results confirm the low genetic diversity found elsewhere in Europe, and no new stable glacial refugium was identified in the easternmost part of its distribution. Nonetheless, we found some closely related but private haplotypes in south of the Dardanelles and in Lesvos island (as some already mentioned by Korsch et al., 2015), suggesting that *T. pityocampa* survived the last glacial period locally in this region. This scenario suggests that *T. pityocampa* is a recent invader in Anatolia sensu Poulakakis et al. (2015). It probably expanded its range in the Eastern Mediterranean in the Pleistocene, long after the MAT had formed. As documented for a diversity of taxa (e.g., Poulakakis et al., 2015), *T. pityocampa* dispersed eastwards from its glacial refugia, circumventing the MAT using a terrestrial corridor, crossed the TSS and spread southward along the coast, finally reaching as south as Chios. Enlarging the study area over the Balkans might allow to determine whether rare haplotypes, such as those found in this study, would occur elsewhere in Europe.

In conclusion, we showed that *T. wilkinsoni* and *T. pityocampa* display a clear parapatric pattern and meet near the TSS in northwestern Turkey. This region corresponds to a hybrid zone between these two organisms, where European *T. pityocampa* recently extended its range and met the northern limit of the western *wilkinsoni* lineage after the last postglacial recolonization. Due to complex paleogeography of the region, where land connections have constantly moved, the TSS could successively act as a geographic barrier between Europe and Anatolia, as for several other terrestrial organisms (e.g., Arntzen & Wielstra, 2010). It could also act as a land bridge allowing species to circumvent the MAT and expand their ranges in either direction (Dubey et al., 2007; Gündüz et al., 2007), facilitating the formation of contact zones (Antoniou, Magoulas, Platis, & Kotoulas, 2013; Bilgin, 2011; Hewitt, 2011).

### 4.2 Characterization of the hybrid zone

#### 4.2.1 The hybrid zone is restricted and mostly bimodal, suggesting limited gene exchange

Locations of contact zones are strongly influenced by biogeographic barriers, which eventually constrain hybrid individuals in certain territories (Barton & Gale, 1993; Kawakami, Butlin, Adams, Paull, & Cooper, 2008). Considering all markers, we here detected a total of 31 hybrid individuals (Table 2), all restricted to a narrow corridor among the Sea of Marmara in the north, Aegean Sea in the west, climatically unsuitable Central Turkey in the east, and İzmir in the south (Figure 5). We did not identify any F_1_, and only found individuals assigned as F_2_ or backcross categories. A similar pattern was found in an *Iris* hybrid zone, in which F_1_ individuals were extremely rare (Sung, Bell, Nice, & Martin, 2018). It should be noted that the analyses of the microsatellite data are not meant to explicitly identify hybridization events that would have occurred more than two generations ago. The individuals identified as backcross or F_2_ by NewHybrids might therefore have a more complex ancestry.

Hybrid zones are usually described according to the uni‐/bimodality of genotypic distribution in the zone. Unimodal hybrid zones are composed of average hybrid genotypes, while bimodal hybrid zones are characterized by excess of individuals resembling either the first or the second parental species and a deficit of average individuals (Jiggins & Mallet, 2000). In the present study, we found mostly pure parents and backcrosses in sites where the corresponding parental species occurred; F_2_ hybrids were only found in Burhaniye (26). Although a more extensive sampling could reveal other patterns, these features, taken together, show that the identified hybrid zone is bimodal sensu Jiggins and Mallet (2000). Overall, the restricted width and general bimodality of the hybrid zone suggest that effective dispersal of genes between the two species is relatively limited. Effective barriers to gene flow, thus, might be at play in the hybrid zone and could be linked to either prezygotic (e.g., assortative mating) or postzygotic (e.g., selection against hybrids) isolation. A similar pattern of limited gene flow was found for the greater mouse‐eared bat in the Thrace region (Furman, Emek, & Çoraman, 2018). In order to understand the nature of this limitation, hybrid fitness (see Bimova et al., 2011) and phenological characteristics of the two species (see Chunco, 2014 and Ording, Mercader, Aardema, & Scriber, 2010) should be specifically investigated in the contact zone.

#### 4.2.2 Discordance between markers reveals direction of gene flow and possible past geographic distributions

Discordance between markers corresponds to situations where a single individual is assigned to one species by some of the loci, whereas it bears allele(s) corresponding to the other species at least for one other marker. Discordances can be found either between nuclear markers (nuc‐nuc discordance; i.e., traces of the nuclear genome of one species in a genomic background of the other), or between nuclear and mitochondrial markers (mt‐nuc discordance; i.e., occurrence of a mitochondrial haplotype of one species in an individual assigned to the other species by all or a majority of the nuclear loci used. This genomic mosaic of introgression might emerge because of unequal exchange of genes and density‐dependant processes (Harrison & Larson, 2014), in which genomic traces of the rarest species eroding with time through backcrosses (Currat, Ruedi, Petit, & Excoffier, 2008). Conflicting geographic patterns between mitochondrial and nuclear markers (mt‐nuc discordance) in secondary contact zones can correspond to divergent patterns of gene flow between the two genomes, and thus to sex‐biased dispersal as mtDNA is inherited from the maternal lineage in most animals. They can also sign past hybrid zone movement, the majority of nuclear markers shifting their range while a wake of mtDNA is left behind (Toews & Breslford, 2012), or introgression by the native species in an expanding one (Currat et al., 2008). In the present study, most hybrids were characterized by a general *T. pityocampa* genomic background, and some traces of *T. wilkinsoni* alleles for a minority of nuclear loci. This pattern supports a scenario of stepping‐stone introgression of *T. wilkinsoni* genes into *T. pityocampa* genomes, corresponding to local gene flow by *T. wilkinsoni* male invaders in already established *T. pityocampa* populations, without signs of successful reproduction of *T. wilkinsoni* females locally. This phenomenon seems to have been particularly intense in Burhaniye, where we could not identify any pure parental individual and where all the individuals that we identified as F_2_ had *T. pityocampa* mitochondrial haplotype.

Opposed to the relatively frequent cases of *T. wilkinsoni* nuclear introgression, we found cases of mt‐nuc discordance only in İzmir (30), where eight out of 18 individuals had a *T. pityocampa* mtDNA in a (mostly) *T. wilkinsoni* nuclear genome. This can be due to asymmetric hybridization facilitating crosses between *T. wilkinsoni* males and *T. pityocampa* females, as such a pattern emerges when interspecific gene exchanges tend to occur preferentially in one direction. Due to the lower mobility of females (a few kilometers on average, whereas male dispersal can be ~20 km on average, Battisti et al., 2015), mtDNA exhibits lower gene flow than nuclear markers in the PPM (Simonato et al., 2007). Accordingly, mtDNA introgression can still be detectable even after many generations. On the other hand, recurrent backcrosses with invading *T. wilkinsoni* might eventually lead to an almost complete erosion of the nuclear *T. pityocampa* genome traces in some individuals. This scenario suggests that *T. pityocampa* was once present on the Turkish shore facing Chios Island and have been displaced by *T. wilkinsoni* on the mainland, while it maintained on the nearby islands. A similar situation has been described recently for the crested newts in northwestern Turkey (Wielstra et al., 2017). Complex geographic history of this region, where ancient gulfs and lakes were once present in today's Menderes and Gediz Deltas (Hakyemez, Erkal, & Göktaş, 1999) and made the landscape more fragmented for forest insects, might have influenced past hybridization events and favored *T. wilkinsoni* on the mainland.

## 5 CONCLUSION AND PERSPECTIVES

Our results allow to go far beyond previous knowledge about the evolution of the PPM sister species. They suggest that the two species diverged in allopatry, survived the successive glacial maxima in disjoint refugial areas, and met at the extreme edge of their recolonization routes during the last interglacial. Fine‐scale genetic diversity patterns nonetheless suggest that *T. pityocampa* refugia were certainly present in Anatolia during last glacial period. Even if reproductive isolation probably due to premating barriers is found, hybridization seems to have played a major role in species displacement, in favor of *T. wilkinsoni* that constrained *T. pityocampa* to western Anatolia. The respective roles of differential ecological traits, competition, and genetic interference in shaping the present patterns remain to be elucidated. Hybrid zones were defined as natural laboratories of evolutionary studies by Hewitt (1988), or “windows on evolutionary process” by Harrison (1990). In the case study presented here, hybrid characterization will offer the possibility to explore the mosaic of introgression along the genome concerning selected adaptive genes and to detect genes involved in reproductive isolation, owing to the recent development of genomics resources in the PPM (Gschloessl et al., 2018; Leblois et al., 2018). Monitoring the hybrid zone using systematic sampling will help us understand whether this pattern is stable over time and whether F_1_ can ever be identified in nature. Factors that shape the dynamics of introgression and limit interspecific gene flow in the field (e.g., differential phenology) would be a complementary research topic. Finally, deciphering the ecological niches of both species through species distribution modeling will shed light on the history of their past, current, and future ranges.

## CONFLICT OF INTEREST

The authors declare no conflict of interest.

## AUTHOR CONTRIBUTION

Author contributions: K.İ., C.B., and C.K. conceived the study; K.İ. conducted the fieldwork in Turkey and Cyprus, and A.B. and C.K. provided samples from other countries and collaborators; K.İ., C.B., and L.S. performed laboratory experiments; K.İ., C.B., and C.K. analyzed the data; K.İ. led the writing with assistance from C.K., C.B., and A.B. All authors read and approved the final manuscript.

## Data Availability

Sequence data available from GenBank: Accessions for COI haplotypes MH742427–MH742477, Accessions for Pho alleles MH742478–MH742487. Microsatellite data available from the Dryad Digital Repository: https://doi.org/10.5061/dryad.1vhhmgqpn
